# Fatty liver index and breast cancer risk: age-stratified findings from a prospective electronic health record–based cohort

**DOI:** 10.3389/fonc.2026.1833372

**Published:** 2026-10-26

**Authors:** Yuqi Ma, Mengyuan Xie, Yanni Li, Qing Li, Mingming Liang

**Affiliations:** 1 Department of Infectious Disease and Liver Disease, The Second Hospital of Nanjing, Affiliated to Nanjing University of Chinese Medicine, Jiangsu, China; 2 Anqing Medical Center of Anhui Medical University, Anqing, Anhui, China; 3 Tuberculosis Prevention and Control Institute, Anhui Provincial Center for Disease Control and Prevention, Hefei, Anhui, China; 4 Department of Obstetrics and Gynecology, Anqing Municipal Hospital, Anqing, Anhui, China; 5 School of Health Management, Anhui Medical University, Hefei, Anhui, China

**Keywords:** fatty liver index, breast cancer, risk factors, modified poisson regression, cox proportional hazards

## Abstract

**Background:**

The fatty liver index(FLI) is a validated noninvasive marker derived from routinely collected clinical parameters. It is widely used to assess hepatic steatosis and metabolic dysfunction. These conditions have been linked to cancer development. The association between the FLI and breast cancer risk remains limited.

**Methods:**

We conducted a prospective analysis of a population based cohort with linked electronic health records. We classified participants into low, intermediate, and high FLI groups. We used modified Poisson regression with robust variance to estimate relative risks of incident breast cancer across index categories. We applied Cox proportional hazards models to estimate hazard ratios while adjusting for demographic, metabolic, lifestyle, and reproductive factors. Lifestyle factors were measured using electronic biosensing devices. We used restricted cubic spline models to evaluate non linear dose response relationships. We also performed analyses stratified by age and menopausal status. Multiple sensitivity analyses assessed the stability of the results.

**Results:**

During follow-up, higher FLI levels were consistently associated with an increased incidence of breast cancer. Compared with women in the low FLI group, those in the intermediate FLI group had an adjusted RR of 1.09 (95% CI, 1.02–1.16) and an HR of 1.08 (95% CI, 1.01–1.15), while those in the high FLI group had an adjusted RR of 1.11 (95% CI, 1.01–1.21) and an HR of 1.11 (95% CI, 1.02–1.21). Restricted cubic spline analyses demonstrated a non-linear association, with a steeper increase in breast cancer risk at higher FLI values. Associations were more pronounced among older and postmenopausal women.

**Conclusions:**

An elevated FLI derived from routinely available electronic health record data is independently associated with a modest increase in breast cancer risk. This association is more evident among older and postmenopausal women. The FLI may serve as a practical marker for breast cancer risk stratification in population health settings. Further research is needed to clarify underlying mechanisms and to evaluate prevention strategies informed by the FLI.

## Introduction

Breast cancer remains the most common malignancy and a leading cause of cancer-related death among women worldwide (1). Despite well-established risk factors such as reproductive history, exogenous hormone exposure, obesity, and alcohol consumption, a considerable proportion of breast cancer cases cannot be explained by these factors alone (1–3). This observation highlights the need to identify additional metabolic and systemic determinants that may contribute to breast cancer development, particularly those that can be assessed using routinely collected health data.

The fatty liver index (FLI) is a noninvasive and cost-effective composite marker that reflects hepatic fat accumulation and broader metabolic dysfunction. It integrates routinely measured clinical parameters, including body mass index, waist circumference, triglycerides, and gamma-glutamyltransferase, and can be readily derived from electronic health records. FLI captures key components of metabolic syndrome, including central obesity, dyslipidemia, impaired liver function, and insulin resistance, which are increasingly recognized as contributors to systemic carcinogenic processes (4). Such metabolic disturbances have been increasingly associated with a higher incidence of extrahepatic cancers.

Growing epidemiological evidence suggests that metabolic dysfunction characterized by hepatic involvement is associated with an increased risk of several extrahepatic malignancies. Several biological mechanisms provide plausible pathways linking metabolic dysfunction indicated by elevated FLI to breast cancer. Insulin resistance and compensatory hyperinsulinemia may promote mitogenic signaling, while chronic low-grade systemic inflammation driven by metabolic abnormalities contributes to the release of pro-tumorigenic cytokines (5, 6). In addition, altered hepatic metabolism influences estrogen synthesis and clearance, which may increase estrogen bioavailability and is particularly relevant for hormone-sensitive breast cancers (7). These mechanistic clues suggest that metabolic dysfunction with hepatic involvement, as assessed by indices such as FLI, could be an important but underexplored determinant of breast cancer risk.

The FLI, derived from body mass index, waist circumference, triglycerides, and gamma-glutamyltransferase, is a validated proxy for hepatic involvement in metabolic syndrome and has been widely applied in large-scale epidemiological studies (8). Prior research has linked higher FLI values to cardiometabolic diseases and several cancer types (9, 10).However, evidence specifically linking FLI to breast cancer risk remains sparse and inconsistent. Most prior studies have relied on limited sample sizes or cross-sectional designs and have not fully explored potential heterogeneity across clinically relevant subgroups.

Age and menopause represent two important determinants of breast cancer biology (11, 12). Incidence rates rise steeply after menopause, coinciding with metabolic changes and increased NAFLD prevalence in older women. Menopausal transition alters sex hormone profiles, modifies fat distribution, and affects hepatic lipid metabolism, all of which may amplify the oncogenic potential of NAFLD. Accordingly, it is biologically plausible that the association between FLI and breast cancer may be more pronounced in older and postmenopausal women.

To address these gaps, we conducted a prospective analysis within a large population-based cohort with linked electronic health records. The present study aimed to examine the association between FLI and incident breast cancer risk and to characterize potential dose–response relationships. We further aimed to assess whether this association differs by age and menopausal status, thereby providing age-stratified evidence on the role of metabolic liver dysfunction in breast cancer development.

## Methods

### Study population

This study was conducted using data from the UK Biobank, a large prospective cohort comprising more than 500,000 participants aged 40–69 years who were recruited between 2006 and 2010 across the United Kingdom (13). The UK Biobank integrates questionnaire data, clinical assessments, electronic health records, and measurements obtained using electronic biosensing devices. For the present analysis, we included women who had complete information on baseline fatty liver index (FLI) components and relevant covariates. Participants with a history of any cancer at baseline, those with missing follow-up data, or those who withdrew consent were excluded. Each participant’s unique identifier (eid) ensured accurate data matching and deduplication. The baseline assessment date was used as the entry point, and follow-up time was calculated until the date of breast cancer diagnosis, death, or end of follow-up, whichever occurred first.

### Exposure assessment

The primary exposure variable was the fatty liver index (FLI), calculated using body mass index (BMI), waist circumference, serum triglycerides, and gamma-glutamyltransferase (GGT) according to a validated algorithm (14). Anthropometric measurements including body mass index and waist circumference were obtained during baseline assessment using standardized electronic measurement devices operated by trained staff. Blood samples were collected at baseline, and biochemical markers including triglycerides and gamma-glutamyltransferase were analyzed in centralized laboratories following standardized protocols.

After downloading and quality-controlling measurements of triglycerides, BMI, GGT and waist circumference from the biobank (ensure triglycerides in mg/dL, GGT in U/L, waist in cm and BMI in kg/m², and handle missing values/outliers), assemble a clean dataset for analysis.

Compute the Fatty Liver Index (FLI) using the formula:

FLI = [exp(0.953·ln(triglycerides) + 0.139·BMI + 0.718·ln(GGT) + 0.053·waist − 15.745)/(1 + exp(0.953·ln(triglycerides) + 0.139·BMI + 0.718·ln(GGT) + 0.053·waist − 15.745))] × 100,

where ln denotes the natural logarithm.

The FLI provides a non-invasive, cost-effective, and easily obtainable surrogate marker for hepatic steatosis. By integrating anthropometric and biochemical parameters, it allows for a reliable estimation of fatty liver risk in large-scale epidemiological and clinical studies without the need for imaging or liver biopsy. FLI was analyzed both as a continuous variable and as categorical groups: low risk (<30), intermediate risk (30–59), and high risk (≥60) (14).

### Outcome ascertainment

The study outcome was incident breast cancer, identified through linkage with national cancer registries using International Classification of Diseases (ICD-10) codes. Incident cases were defined as first-time diagnoses of breast cancer occurring after baseline. Participants without a breast cancer diagnosis during follow-up were censored at the date of death, loss to follow-up, or the administrative end of follow-up.

### Covariates

We selected potential confounders based on prior literature and biological relevance. Demographic variables included baseline age, ethnic background, education level, and the Townsend deprivation index. Lifestyle related factors were derived from a combination of self reported questionnaires and measurements obtained using electronic biosensing devices. Smoking status and alcohol consumption were assessed by standardized questionnaires administered at baseline.

Physical activity was quantified using data from wearable accelerometer based devices in a subset of participants, complemented by questionnaire based assessments of moderate and vigorous activity. Sleep duration was assessed using self reported data, with objective sleep related metrics derived from wrist worn electronic devices where available. Health related factors included body mass index, diabetes status, and menopausal status. Hormone replacement therapy use was recorded through baseline questionnaires and linked prescription records. All covariates were incorporated into multivariable models to reduce residual confounding.

### Statistical analysis

Baseline characteristics across FLI groups were compared using the Kruskal–Wallis test for continuous variables and chi-squared tests for categorical variables. Relative risks (RRs) and 95% confidence intervals (CIs) for breast cancer occurrence during follow-up were estimated using log-binomial regression; when models failed to converge, modified Poisson regression with robust error variance was applied (15). Models were adjusted sequentially for age, and then additionally for BMI, diabetes, menopausal status, hormone replacement therapy (HRT), smoking, alcohol intake, physical activity, and sleep duration.

The primary analysis used Cox proportional hazards models to estimate hazard ratios (HRs) and 95% CIs for breast cancer incidence across FLI categories, with the low-FLI group as reference. Four sequential models were fitted: unadjusted (Model 0), adjusted for demographics (Model 1), lifestyle factors (Model 2), and additionally for BMI, diabetes, menopausal status, and HRT use (Model 3). FLI was examined both as categorical and continuous exposure. Non-linear associations between continuous FLI and breast cancer risk were examined using restricted cubic spline (RCS) functions with five knots at the 5th, 25th, 50th, 75th, and 95th percentiles of the FLI distribution in fully adjusted Cox models. Non-linearity was assessed using likelihood ratio tests.

Pre-specified subgroup analyses were conducted stratified by age category and menopausal status, and multiplicative interaction terms were included to formally test for effect modification. Sensitivity analyses included modeling FLI as a continuous variable; and excluding participants with breast cancer at baseline. All analyses were conducted using R software, version 4.5.0 (R Foundation for Statistical Computing, Vienna, Austria), with two-sided p-values <0.05 indicating statistical significance.

## Results

### Baseline characteristics

Among 218,786 women included in the analysis, 7,699 incident breast cancer cases were recorded during follow-up (**Supplementary Figure S1**; **Table 1**). The median age was 57 years, and the median FLI was 27.8 (IQR: 11.8–58.8). Women with higher FLI were older and displayed a more adverse metabolic profile. Specifically, the median BMI increased from 23.6 in the low-FLI group to 32.5 in the high-FLI group (p < 0.001). The prevalence of diabetes rose from 1.2% in the low-FLI group to 8.8% in the high-FLI group (p < 0.001). Similarly, the proportion of women with postmenopausal status increased across FLI categories, reaching 62% in the high-FLI group compared with 56% in the low-FLI group. Lifestyle differences were also evident: women with higher FLI were less likely to engage in frequent physical activity, reported shorter sleep duration, and had a slightly higher prevalence of current smoking and alcohol abstinence. Notably, breast cancer incidence increased gradually with higher FLI, from 3.3% in the low group to 3.9% in the high group (p < 0.001).

**Table 1:** Baseline characteristics of study participants by FLI groups.

Variable	Overall N = 218,7861	Low risk N = 114,5671	Intermediate risk N = 51,1831	High risk N = 53,0361	P-value2
Age	57.0 (50.0, 63.0)	55.0 (48.0, 62.0)	59.0 (52.0, 64.0)	59.0 (52.0, 63.0)	<0.001
BMI	25.9 (23.3, 29.5)	23.6 (21.9, 25.2)	27.5 (26.0, 29.2)	32.5 (30.0, 35.8)	<0.001
FLI	27.8 (11.8, 58.8)	12.4 (7.1, 19.5)	43.1 (36.1, 51.1)	80.6 (70.1, 90.7)	<0.001
SES	-2.2 (-3.7, 0.3)	-2.4 (-3.8, -0.1)	-2.3 (-3.7, 0.2)	-1.8 (-3.4, 1.1)	<0.001
BMI_group					<0.001
<25	87,674/218,786 (40%)	80,868/114,567 (71%)	6,375/51,183 (12%)	431/53,036 (0.8%)	
25-29.9	81,208/218,786 (37%)	33,001/114,567 (29%)	35,816/51,183 (70%)	12,391/53,036 (23%)	
>=30	49,904/218,786 (23%)	698/114,567 (0.6%)	8,992/51,183 (18%)	40,214/53,036 (76%)	
Moderate physical activity					<0.001
0 days	28,052/218,786 (13%)	11,671/114,567 (10%)	6,745/51,183 (13%)	9,636/53,036 (18%)	
1–2 days	49,595/218,786 (23%)	25,174/114,567 (22%)	11,773/51,183 (23%)	12,648/53,036 (24%)	
3–4 days	57,897/218,786 (26%)	31,212/114,567 (27%)	13,545/51,183 (26%)	13,140/53,036 (25%)	
>=5 days	83,242/218,786 (38%)	46,510/114,567 (41%)	19,120/51,183 (37%)	17,612/53,036 (33%)	
Sleep					<0.001
<6h	11,683/218,786 (5.3%)	4,885/114,567 (4.3%)	2,842/51,183 (5.6%)	3,956/53,036 (7.5%)	
6-7h	123,996/218,786 (57%)	66,811/114,567 (58%)	28,450/51,183 (56%)	28,735/53,036 (54%)	
8h	66,354/218,786 (30%)	35,651/114,567 (31%)	15,668/51,183 (31%)	15,035/53,036 (28%)	
>8h	16,753/218,786 (7.7%)	7,220/114,567 (6.3%)	4,223/51,183 (8.3%)	5,310/53,036 (10%)	
Smoking					<0.001
Previous	69,002/218,786 (32%)	33,810/114,567 (30%)	16,773/51,183 (33%)	18,419/53,036 (35%)	
Never	130,471/218,786 (60%)	71,168/114,567 (62%)	29,781/51,183 (58%)	29,522/53,036 (56%)	
Current	18,708/218,786 (8.6%)	9,319/114,567 (8.1%)	4,470/51,183 (8.7%)	4,919/53,036 (9.3%)	
Prefer not to answer	605/218,786 (0.3%)	270/114,567 (0.2%)	159/51,183 (0.3%)	176/53,036 (0.3%)	
Alcohol					<0.001
Previous	7,506/218,786 (3.4%)	3,313/114,567 (2.9%)	1,656/51,183 (3.2%)	2,537/53,036 (4.8%)	
Never	11,316/218,786 (5.2%)	4,919/114,567 (4.3%)	2,735/51,183 (5.3%)	3,662/53,036 (6.9%)	
Current	199,831/218,786 (91%)	106,280/114,567 (93%)	46,748/51,183 (91%)	46,803/53,036 (88%)	
Prefer not to answer	133/218,786 (<0.1%)	55/114,567 (<0.1%)	44/51,183 (<0.1%)	34/53,036 (<0.1%)	
Education					<0.001
High school	10,111/196,801 (5.1%)	5,551/104,581 (5.3%)	2,350/45,547 (5.2%)	2,210/46,673 (4.7%)	
Higher	32,507/196,801 (17%)	19,697/104,581 (19%)	6,829/45,547 (15%)	5,981/46,673 (13%)	
Missing	1,501/196,801 (0.8%)	647/104,581 (0.6%)	405/45,547 (0.9%)	449/46,673 (1.0%)	
None	31,499/196,801 (16%)	12,162/104,581 (12%)	8,692/45,547 (19%)	10,645/46,673 (23%)	
Secondary	121,183/196,801 (62%)	66,524/104,581 (64%)	27,271/45,547 (60%)	27,388/46,673 (59%)	
Diabetes					<0.001
No	210,985/218,786 (96%)	113,050/114,567 (99%)	49,738/51,183 (97%)	48,197/53,036 (91%)	
Yes	7,419/218,786 (3.4%)	1,391/114,567 (1.2%)	1,360/51,183 (2.7%)	4,668/53,036 (8.8%)	
HRT_use					<0.001
No	136,322/218,786 (62%)	76,348/114,567 (67%)	29,434/51,183 (58%)	30,540/53,036 (58%)	
Yes	81,863/218,786 (37%)	37,963/114,567 (33%)	21,587/51,183 (42%)	22,313/53,036 (42%)	
Menopause					<0.001
No	54,771/218,786 (25%)	35,836/114,567 (31%)	9,656/51,183 (19%)	9,279/53,036 (17%)	
Yes	130,190/218,786 (60%)	63,990/114,567 (56%)	33,147/51,183 (65%)	33,053/53,036 (62%)	
Breast Cancer Event	7,699/218,786 (3.5%)	3,734/114,567 (3.3%)	1,901/51,183 (3.7%)	2,064/53,036 (3.9%)	<0.001

1Median (Q1, Q3); n/N (%).

2Kruskal-Wallis rank sum test; Pearson’s Chi-squared test.

SES, Socioeconomic Status.

### Breast cancer incidence by FLI category

Before the regression analyses, we examined crude breast cancer incidence across FLI categories. Breast cancer events occurred in 3,734 of 114,567 women (3.3%) in the low FLI group, 1,901 of 51,183 women (3.7%) in the intermediate FLI group, and 2,064 of 53,036 women (3.9%) in the high FLI group (**Table 1**). This simple comparison showed a gradual increase in incidence across FLI categories.

### Modified Poisson regression

Modified Poisson regression models examining the cross-sectional association between FLI categories and breast cancer showed results consistent with prospective analyses (**Figure 1**; **Supplementary Table S1**). Compared with women in the low-FLI group, those with intermediate and high FLI had significantly higher risk of breast cancer. After multivariable adjustment, the relative risks (RRs) were 1.09 (95% CI: 1.02–1.16) for intermediate FLI and 1.11 (95% CI: 1.01–1.21) for high FLI. Stratified analyses indicated that the association was stronger in older women (≥60 years: RR = 1.09, 95% CI: 1.00–1.20 for intermediate FLI; RR = 1.03, 95% CI: 0.90–1.17 for high FLI) and in postmenopausal women (RR = 1.10, 95% CI: 1.02–1.19 for intermediate FLI; RR = 1.12, 95% CI: 1.01–1.25 for high FLI), whereas associations were weaker and non-significant among younger or premenopausal women.

**Figure 1:**
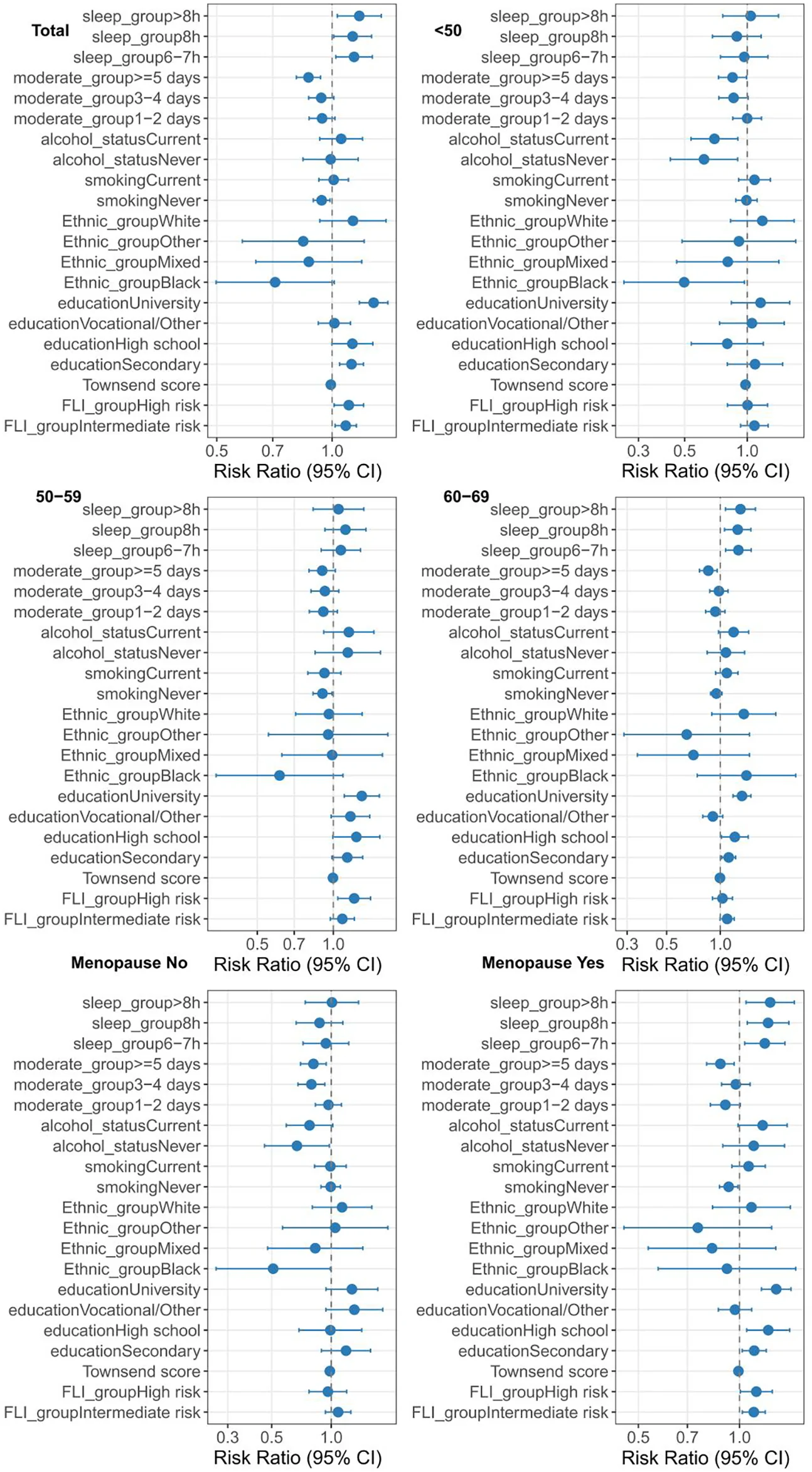
Risk ratios (RRs) for breast cancer across categorical FLI groups from modified Poisson regression models. The reference group was the low FLI category. The fully adjusted model included baseline age, Townsend deprivation index, education, ethnicity, smoking, alcohol intake, physical activity, sleep duration, BMI, diabetes, HRT use, and menopausal status. Error bars indicate 95% confidence intervals.

In Cox regression analyses (**Figure 2**; **Table 2**), elevated FLI was consistently associated with increased risk of incident breast cancer. In unadjusted models, women in the intermediate- and high-FLI groups had 15% and 22% higher risks, respectively, compared with the low-FLI group. After adjusting for demographics, lifestyle factors, BMI, diabetes, HRT use, and menopausal status, the associations remained significant though attenuated: HR = 1.08 (95% CI: 1.01–1.15; p = 0.017) for intermediate FLI and HR = 1.11 (95% CI: 1.02–1.21; p = 0.020) for high FLI. Model concordance improved from 0.723 in the unadjusted model to 0.762 in the fully adjusted model, indicating incremental explanatory power with covariate adjustment.

**Figure 2:**
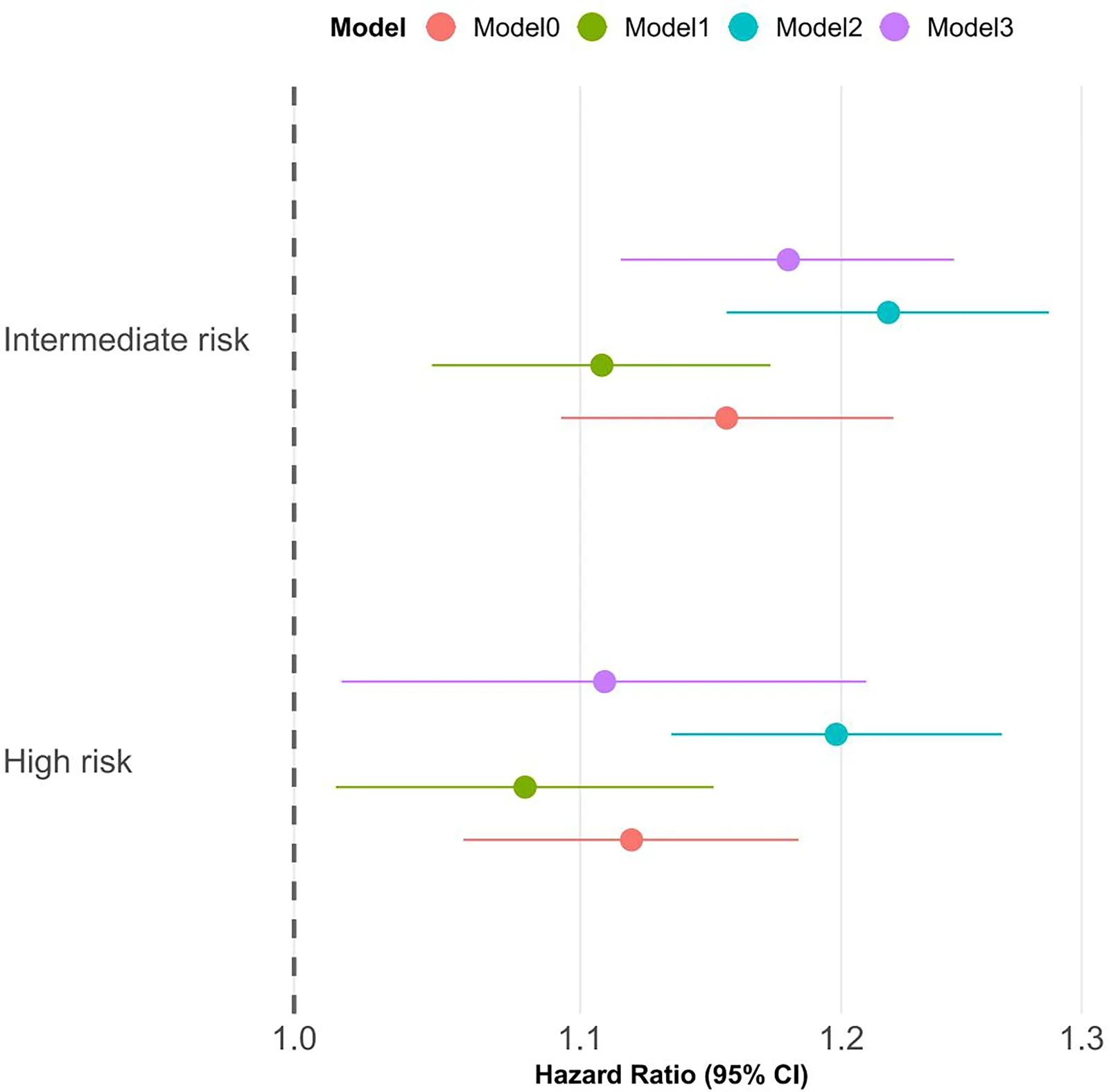
Hazard ratios (HRs) from Cox proportional-hazards models for FLI and incident breast cancer.

**Table 2:** Cox proportional hazards models for the association between FLI group and outcome.

Variable	Model 0 (unadjusted) n = 218,786; events = 7,699	Model 1 (demographics)	Model 2 (+ lifestyle)	Model 3 (fully variables.)
FLI — Intermediate risk (vs Low)	1.155 (1.093–1.221); p < 0.001	1.119 (1.058–1.183); p < 0.001	1.108 (1.047–1.172); p < 0.001	1.080 (1.014–1.150); p = 0.017
FLI — High risk (vs Low)	1.219 (1.155–1.286); p < 0.001	1.198 (1.134–1.266); p < 0.001	1.179 (1.115–1.246); p < 0.001	1.109 (1.016–1.210); p = 0.020

Model definitions:.

Model 0 = unadjusted;.

Model 1 = adjusted for baseline_age, Townsend deprivation index, education, Ethnic;.

Model 2 = Model 1 + smoking, alcohol, physical activity, sleep;.

Model 3 = Model 2 + BMI, diabetes, HRT, menopause.

Concordance: Model0 = 0.723; Model1 = 0.757; Model2 = 0.761; Model3 = 0.762.

Likelihood ratio test p-values for all models: p < 2e-16 (see Methods for details).

Reference group: FLI = Low risk. Subgroup analyses.

Age-stratified analyses highlighted heterogeneity in the association between FLI and breast cancer risk (**Figure 3**). In age-stratified Cox proportional hazards models, higher FLI was associated with increased breast cancer risk in most age groups. Among women aged <50 years, the HR was 1.61 (95% CI: 0.95–2.70; p = 0.075) for intermediate FLI and 1.82 (95% CI: 1.09–3.03; p = 0.021) for high FLI. In women aged 50–59 years, the corresponding HRs were 1.12 (95% CI: 1.00–1.26; p = 0.052) and 1.28 (95% CI: 1.14–1.43; p < 0.001), while in those aged 60–69 years, the HRs were 1.17 (95% CI: 1.07–1.28; p < 0.001) and 1.28 (95% CI: 1.17–1.40; p < 0.001) for intermediate and high FLI, respectively. No statistically significant associations were observed in women aged ≥70 years. Menopausal status further modified the relationship: in postmenopausal women, high FLI was associated with a 17% increased risk of breast cancer (HR = 1.17, 95% CI: 1.01–1.31), whereas no significant association was observed in premenopausal women. These findings suggest that aging and menopausal transition may amplify the carcinogenic impact of metabolic liver dysfunction.

**Figure 3:**
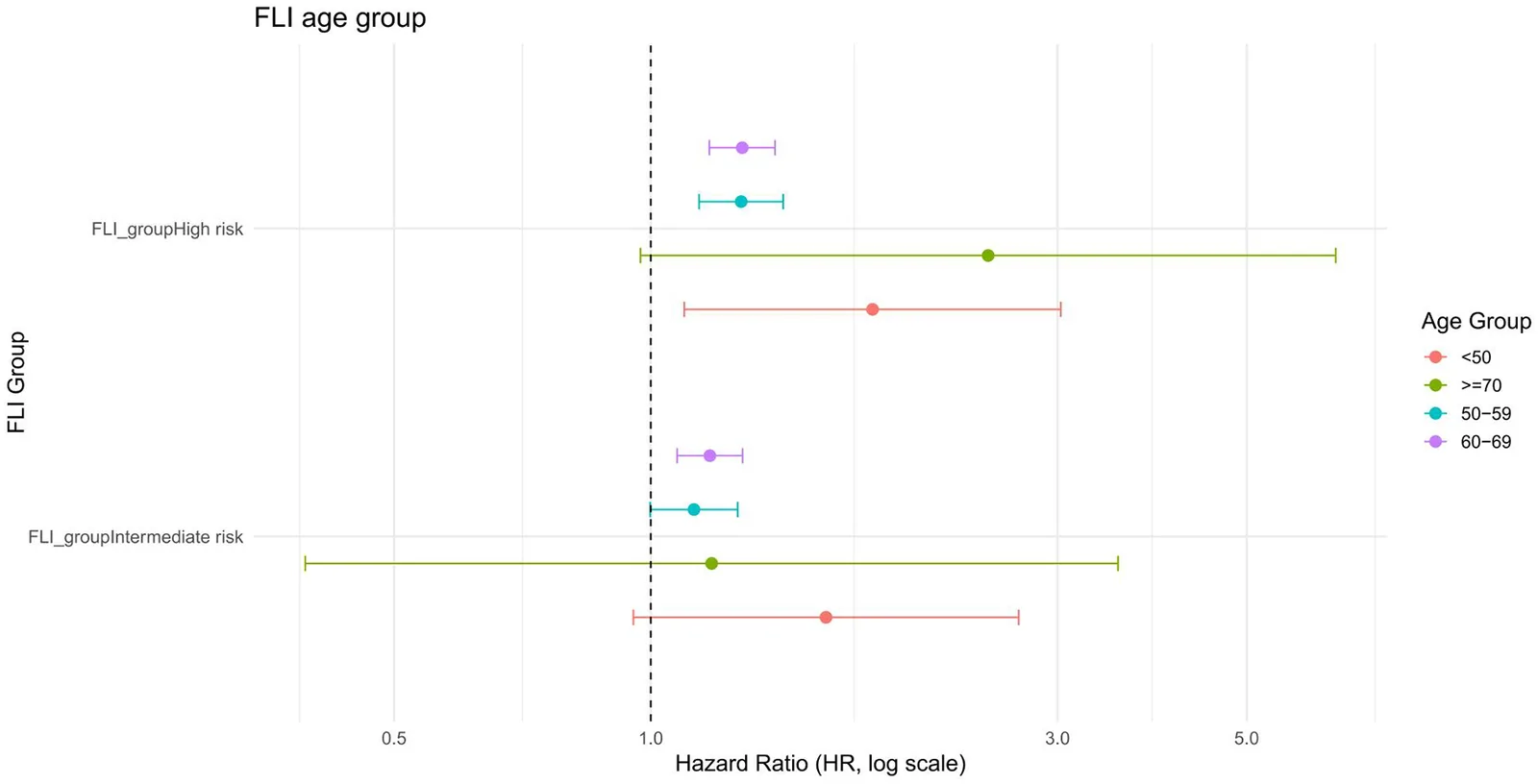
Subgroup analyses: multivariable-adjusted Cox HRs for FLI and breast cancer stratified by key covariates.

### Dose–response relationship

Restricted cubic spline analyses revealed a non-linear dose–response relationship between FLI and breast cancer risk (**Figure 4**). At very low FLI levels (<25), breast cancer risk was slightly below unity, suggesting a modest protective association. Risk increased progressively at FLI values above 50, with the curve plateauing around an HR of the highest values. The confidence intervals widened at the extreme tail, reflecting fewer observations at very high FLI. Overall, the spline model supported a threshold effect, where risk elevation became more evident at higher FLI levels and did not increase linearly across the full range.

**Figure 4:**
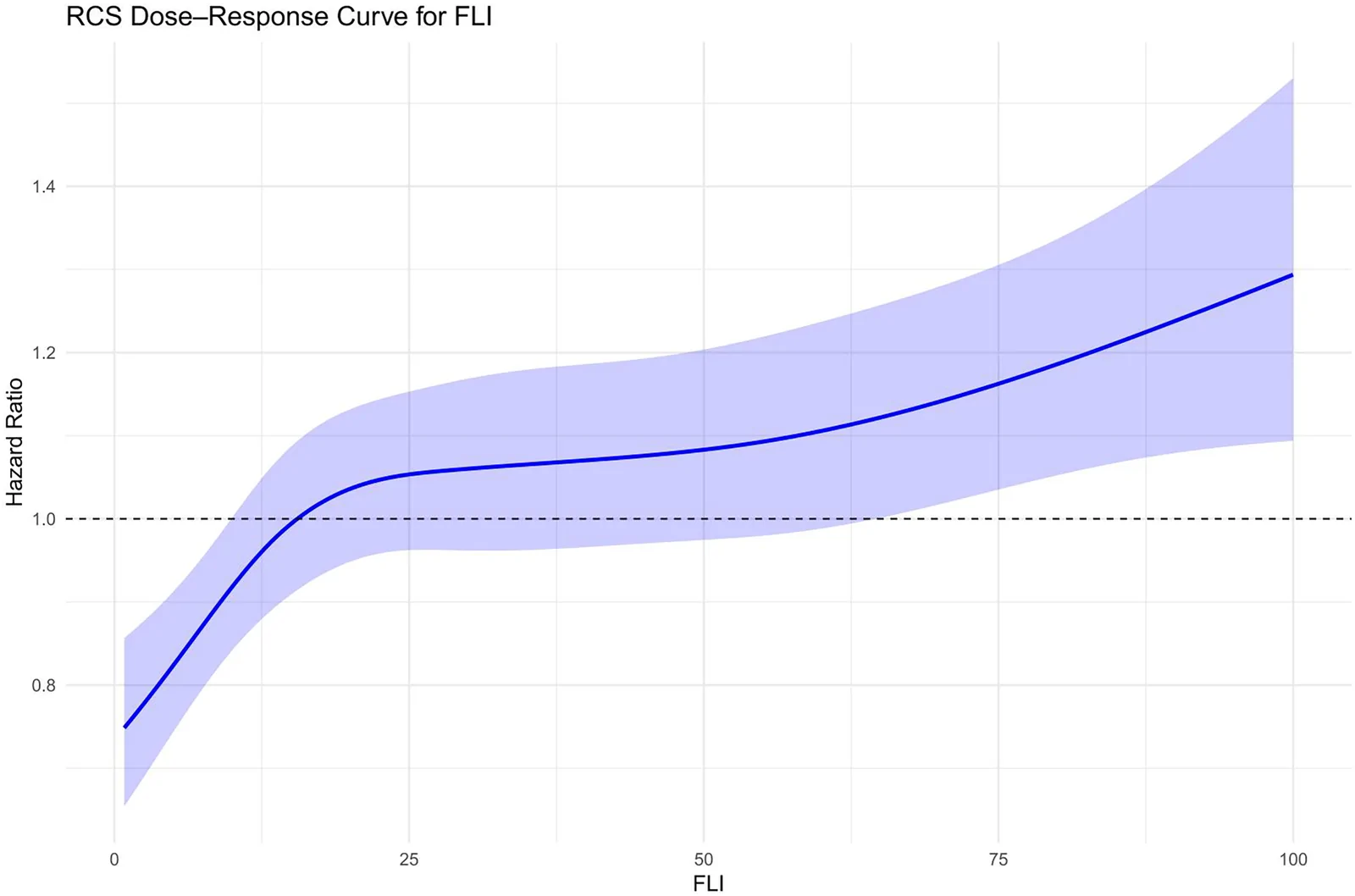
|Restricted cubic spline showing the dose–response relationship between continuous FLI and breast cancer risk.

### Sensitivity analyses

Sensitivity analyses treating FLI as a continuous variable yielded consistent results (**Supplementary Table S2**). Each unit increase in FLI was associated with a modest but statistically significant rise in breast cancer risk (HR per unit = 1.003, 95% CI: 1.002–1.004; p < 0.001), with similar estimates across different adjustment models. Exclusion of breast cancer cases diagnosed within the first two years of follow-up did not materially alter the findings. Further adjustment for additional lifestyle variables, including alcohol frequency and physical activity, produced results consistent with the main analyses.

## Discussion

In this large, prospective cohort of women, we demonstrated that higher FLI levels were associated with an increased risk of breast cancer. The lifestyle related covariates were derived from measurements obtained using electronic biosensing devices, which provided objective and standardized assessments beyond self reported data. The integration of device based measurements, including physical activity and sleep related metrics, reduced recall bias and improved the precision of exposure characterization. Such objective assessment is particularly relevant in studies of metabolic health, where subtle behavioral patterns may influence hepatic function and cancer risk through cumulative physiological effects. Using both modified Poisson regression for relative risk estimation and Cox proportional hazards models for time-to-event analyses, we consistently observed a positive association, particularly among older and postmenopausal women. Importantly, the non-linear dose–response pattern suggested a threshold effect, with substantially elevated risk appearing at higher FLI levels. These findings indicate that metabolic liver dysfunction may be associated with breast cancer risk beyond its role as a comorbidity.

Several plausible biological pathways may explain the observed association. FLI reflects hepatic fat accumulation and systemic metabolic dysfunction, characterized by insulin resistance, hyperinsulinemia, dyslipidemia, and chronic low-grade inflammation (5, 6). These metabolic derangements can enhance mitogenic signaling through insulin and insulin-like growth factor-1 pathways, promote oxidative stress, increase reactive oxygen species production, and alter estrogen metabolism, all of which have been implicated in breast carcinogenesis (16–18). Chronic hepatic inflammation may further upregulate pro-inflammatory cytokines and adipokines, fostering a tumor-promoting microenvironment (17, 18). In particular, postmenopausal women may be more susceptible to these pro-tumorigenic effects via peripheral aromatization of androgens in adipose tissue and altered hormone-binding globulin levels (19).

The threshold-like risk pattern we observed may reflect a tipping point beyond which the accumulation of metabolic insults overwhelms compensatory mechanisms, thereby accelerating tumor initiation and progression (20). This is consistent with experimental models showing that steatosis-associated oxidative stress and endoplasmic reticulum stress trigger DNA damage and epigenetic modifications that may promote malignant transformation (21).

Our results align with and extend existing evidence linking NAFLD and metabolic syndrome to increased breast cancer risk (16). Previous cohort studies often relied on physician-diagnosed NAFLD or imaging-based assessments, which may limit applicability in large-scale screening. By contrast, FLI is derived from routine anthropometric and biochemical indicators, enabling efficient risk stratification in population-based settings. While prior studies have variably reported that the association between NAFLD and breast cancer risk is stronger in postmenopausal women (19, 22), our findings clarify that the risk elevation is particularly pronounced in women aged 60–69 years, suggesting a combined influence of metabolic aging and hormonal changes.

This study contributes to the growing body of literature on metabolic dysfunction and cancer risk by focusing on FLI as a readily accessible biomarker. Unlike traditional metabolic syndrome criteria, FLI integrates hepatic and systemic metabolic indicators into a single score, capturing dimensions of metabolic health often overlooked in cancer epidemiology. By applying both relative risk models and time-to-event Cox analyses, our study showed consistent associations across different epidemiological modeling frameworks.

The large sample size and detailed adjustment for lifestyle, reproductive, and hormonal factors support the stability of the observed association in an area where metabolic health and cancer risk remain insufficiently disentangled. Because FLI can be calculated from routinely collected measures, it could be integrated into standard health check-ups and preventive care programs. From a public health perspective, the global rise in metabolic dysfunction signals an urgency to broaden breast cancer prevention beyond traditional reproductive, lifestyle, and genetic risk factors, incorporating metabolic and hepatic health as modifiable targets.

Our subgroup findings highlight that metabolic liver dysfunction may have particularly detrimental effects in older and postmenopausal women, supporting the development of age-specific and menopausal-status–specific risk prediction tools. In resource limited settings, where imaging based NAFLD screening is not feasible, incorporating FLI into risk assessment models could aid in prioritizing women for lifestyle intervention and surveillance.

The major strengths of this study include its prospective design, large and well-characterized cohort, standardized calculation of FLI, and application of complementary epidemiological models. The use of restricted cubic spline analyses allowed us to identify a non-linear, threshold-like risk pattern. Nonetheless, some limitations must be acknowledged. First, FLI is a surrogate marker that does not capture histological features such as fibrosis or inflammation severity, and misclassification is possible. Second, residual confounding from unmeasured factors, including detailed dietary composition or genetic polymorphisms affecting lipid metabolism, cannot be entirely excluded. Third, as an observational study, causal inference remains limited despite careful adjustment and sensitivity analyses. Fourth, inflammatory markers such as high-sensitivity C-reactive protein (hs-CRP) were not included, which limited assessment of whether systemic inflammation added predictive information beyond FLI. hs-CRP is an important and clinically accessible marker of chronic low-grade systemic inflammation and may provide complementary information to FLI, which primarily reflects metabolic and hepatic dysfunction. Nevertheless, the absence of hs-CRP data does not undermine the present findings or alter our primary conclusion that higher FLI was associated with a modestly increased incidence of breast cancer, particularly among older and postmenopausal women.

Future research should evaluate whether longitudinal changes in FLI predict dynamic shifts in breast cancer risk, and whether targeted interventions to improve hepatic and metabolic health can reduce this risk. Integrating FLI into multi-marker predictive models alongside hs-CRP, genetic and hormonal profiles may further enhance individualized risk assessment. Additionally, mechanistic studies are warranted to delineate the molecular pathways linking hepatic steatosis to mammary tumorigenesis, particularly in the context of aging and menopause.

## Conclusion

In conclusion, elevated FLI is independently associated with a higher risk of breast cancer, particularly in older and postmenopausal women. These findings provide epidemiological evidence linking comprehensive metabolic dysfunction, including central obesity, dyslipidemia, and insulin resistance, with breast carcinogenesis. FLI could serve as a clinical tool for patient risk assessment and as a public health measure for population-level risk stratification. Future studies should validate these results in diverse populations. They should also investigate whether improving metabolic health, including reducing liver fat, can help lower breast cancer risk.

## Data Availability

The data analyzed in this study is subject to the following licenses/restrictions: The data used in this study were obtained from the UK Biobank (Project ID: 151711). Researchers may apply for access to the data through the UK Biobank official website: https://www.ukbiobank.ac.uk/. Requests to access these datasets should be directed to The data used in this study were obtained from the UK Biobank (Project ID: 151711). Researchers may apply for access to the data through the UK Biobank official website: https://www.ukbiobank.ac.uk/.
