# Supplementary material for: Fatty liver index and breast cancer risk: age-stratified findings from a prospective electronic health record–based cohort
Source: Front Oncol. 2026 Oct 26;16:1833372. doi: 10.3389/fonc.2026.1833372 (PMC13745251; doi:10.3389/fonc.2026.1833372)
Supplement: Supplementary file 1 [file DataSheet1.docx]

Supplement files

Table S1 Risk Ratios and 95% Confidence Intervals (CIs) of Breast Cancer Incidence

| Group | RR (95% CI) Total | RR (95% CI) Age Group 1 | RR (95% CI) Age Group 2 | RR (95% CI) Age Group 3 | RR (95% CI) Premenopausal | RR (95% CI) Postmenopausal |
| --- | --- | --- | --- | --- | --- | --- |
| FLI: High Risk | 1.11 (1.01–1.21) | 1.00 (0.80–1.25) | 1.21 (1.04–1.41) | 1.03 (0.90–1.17) | 0.96 (0.77–1.20) | 1.12 (1.01–1.25) |
| FLI: Intermediate Risk | 1.09 (1.02–1.16) | 1.08 (0.93–1.26) | 1.09 (0.97–1.21) | 1.09 (1.00–1.20) | 1.08 (0.93–1.26) | 1.10 (1.02–1.19) |

Table S2 Hazard Ratios for FLI and Breast Cancer Risk in Sensitivity Models

| Model | CovariatesAdjusted | HR(95%CI) | p-value |
| --- | --- | --- | --- |
| cox0 | Unadjusted | 1.003 (1.003–1.004) | <0.001 |
| cox1 | Age, Townsend index, Education, Ethnicity | 1.003 (1.002–1.004) | <0.001 |
| cox2 | Age, Townsend, Education, Ethnicity, Smoking, Alcohol, Physical activity, Sleep | 1.003 (1.002–1.004) | <0.001 |
| cox3 | Age, Townsend, Education, Ethnicity, Smoking, Alcohol, Physical activity, Sleep, BMI, Diabetes, HRT use, Menopause | 1.003 (1.002–1.004) | <0.001 |

Figure S1 Flow diagram of study population selection from the UK Biobank cohort
